# Rutin via Increase in the CA3 Diameter of the Hippocampus Exerted Antidepressant-Like Effect in Mouse Model of Maternal Separation Stress: Possible Involvement of NMDA Receptors

**DOI:** 10.1155/2020/4813616

**Published:** 2020-06-07

**Authors:** Maryam Anjomshoa, Shakiba Nasiri Boroujeni, Sorayya Ghasemi, Zahra Lorigooini, Ahmad Amiri, Shima Balali-dehkordi, Hossein Amini-khoei

**Affiliations:** Medical Plants Research Center, Basic Health Sciences Institute, Shahrekord University of Medical Sciences, Shahrekord, Iran

## Abstract

**Methods:**

Mouse neonates were exposed to MS paradigm 3 hours daily from postnatal days (PND) 2 to 14. The control and MS mice were divided separately into 16 groups (*n* = 8) (8 groups for each set) including mice that received normal saline, mice that received rutin at doses of 10, 50, and 100 mg/kg, mice that received NMDA at a dose of 150 mg/kg, mice that received ketamine (NMDA antagonist) at a dose of 0.25 mg/kg, mice that received NMDA antagonist plus a subeffective dose of rutin, and mice that received NMDA plus an effective dose of rutin. Forced swimming test (FST) was performed. Afterwards, the hippocampus was evaluated in cases of histopathological changes as well as expression of NR2A and NR2B genes.

**Results:**

Rutin significantly reduced immobility time in the FST. The expression of NR2A and NR2B subunits of NMDA receptor in MS mice was significantly higher than that in the control group. Rutin significantly decreased the expression of NR2B and NR2A subunits in the hippocampus. The CA3 diameter and percentage of dark neurons in the hippocampus of MS mice significantly decreased and increased, respectively, which partially reversed following rutin administration.

**Conclusion:**

Rutin, partially, through a neuroprotective effect on the hippocampus exerted antidepressant-like effect. We concluded that NMDA receptors, at least in part, mediated the beneficial effect of rutin.

## 1. Introduction

Depression is a multifactorial, high-economic burden and chronic disease that affects 20% of the world population and is considered as one of the top ten causes of mortality [1, 2]. Current therapies commonly alter the monoamine neurotransmitters in the CNS [3]. However, only about half of the patients show adequate response to these drugs, and many of them are leaving treatment due to side effects [4]. In recent years, researches have been focused on other factors involved in the pathophysiology of depression other than common monoamine pathways [1].

Maternal separation (MS) is an animal model designed to induce stress in the early life [5]. MS is defined as the lack of care, short-term care, or repeated separation from mothers during early life. This type of stress can negatively affect the development of the brain and subsequently lead to impairment in social behavior. Infants under MS stress are prone to development of anxiety, depression, memory loss, and neurological disorders [6–8].

The hippocampus is a part of the brain's limbic system which plays an important role in regulating emotions [9]. Researchers have shown that people with depression have a smaller hippocampal size [10]. Animal studies have also shown that depression induced by exposure to unpredictable chronic stress is associated with a significant increase in the hippocampal CA3 neurodegeneration [11]. Dystrophic lesions of the hippocampal CA3 neurons have been reported in rats with depressive-like behaviors [12].

Glutamate is a major excitatory neurotransmitter in the CNS, which is involved in many physiological conditions such as brain development, synaptic flexibility, memory, and learning [13, 14]. Studies on mice exposed to chronic stress have shown an increase in expression of hippocampal NMDA receptor subunits [15]. For this reason, researchers have considered NMDA receptors for possible therapeutic aspects of depression. In this regard, it has been observed that ketamine and other NMDA antagonists possessed antidepressant effects [16].

It has been well-known that natural compounds have an important role in the management and treatment of depression [17, 18]. In this regard, previous studies have demonstrated that flavonoids could attenuate the depressive-like behaviors in animal models of depression [19, 20]. Rutin, which is also known as rutoside and quercetin 3-O-rutinoside, is a glycosidic compound [21]. Rutin is a flavonoid compound found in many plants, including citrus fruits, Buckwheat, leaves of Rheum species, and Asparagus [22–26]. To date, a wide range of biological activities have been proposed for rutin including reduced permeability and fragility of the capillaries, anti-inflammatory, antioxidant, and neuroprotective activities [22, 27–29]. Preclinical studies have also been shown that rutin has antidepressant effects [11]. Given the abovementioned properties of rutin, as well as the fact that the exact mechanisms of its beneficial effects have not yet been determined, the present study is aimed at investigating the role of NMDA receptors in the antidepressant-like effect of rutin with a focus on histological changes in the hippocampus.

## 2. Material and Methods

### 2.1. Animals

Pregnant NMRI mice (first day of pregnancy) (22–28 g weight) were used (Pasteur Institute, Tehran, Iran). Mice were maintained in standard laboratory conditions including 12 h light/12 h dark, 22 ± 1°C with equal access to food and water.

Day of birth was considered as postnatal day (PND) 0. From PND2, neonates were exposed to the maternal separation paradigm [5] for 3 hours daily until PND14. On PND14, the infants were returned to their mother cages and kept intact until day 21. From day 21, mice were isolated from their mother and were then kept in cages in groups of 4 until PND 60 (22–30 g weight). Control mice were kept in the mother cage from PND0 to PND21 without manipulation and were then kept in cages in groups of 4 from PND21 to PND60.

### 2.2. Drugs

The drugs used in this study were as follows: (1) rutin, (2) ketamine (an NMDA antagonist), and (3) NMDA (as NMDA agonist). All drugs were purchased from Sigma, St Louis, MO, USA. All drugs were dissolved in 0.9% saline in a volume of 10 ml/kg and were administered intraperitoneally (i.p.). The dose of each drug was adjusted according to animal body weight (mg of drug/kg of body weight of mice).

### 2.3. Study Design

128 male NMRI mice aged 60-61 days were divided into 16 groups (*n* = 8). The groups were as follows: group 1: control mice received normal saline at a dose of 10 ml/kg; groups 2, 3, and 4: control mice received rutin at the doses of 10, 50, and 100 mg/kg, respectively; group 5: MS mice received normal saline at a dose of 10 ml/kg; groups 6, 7, and 8: MS mice received rutin at the doses of 10, 50, and 100 mg/kg, respectively; group 9: control mice received NMDA antagonist (ketamine) at a dose of 0.25 mg/kg; group 10: MS group received ketamine at a dose of 0.25 mg/kg; group11: control mice received the NMDA at a dose of 150 mg/kg; group 12: MS mice received the NMDA at a dose of 150 mg/kg; group 13: control mice received ketamine plus a subeffective dose of rutin; group 14: MS group received ketamine plus a subeffective dose of rutin; group15: control mice received the effective dose of rutin plus NMDA; and group 16: MS mice received the effective dose of rutin plus NMDA. We treated mice with NMDA (15 min), ketamine (60 min), and rutin (60 min) prior to the behavioral test. Dose and time of drug administrations were chosen based on previous studies as well as our pilot study [30–32].

### 2.4. Forced Swimming Test (FST)

FST is one of the valid and common tests used for evaluation of depression in rodents. In this experiment, a glass container (12 cm by 25 cm) is filled to a height of 15 cm with water at 25°C. The animal was then gently placed in water. The total compulsory swimming course is 6 minutes, and the first two minutes are considered to match the animal with the new conditions and the immobilization time is recorded for the next 4 minutes [5]. In the FST, an increase in immobility time reflects the inability of mice to deal with an acute unescapable challenge expressing the depressive-like behaviors.

### 2.5. Pathological Assessment

After the behavioral test, animals were killed by high doses of pentobarbital (60 mg/kg, i.p.). Cardiac perfusion was performed with 0.9% normal saline and then with 4% paraformaldehyde in 0.1 ml of cold phosphate buffer (pH = 7.5), and then, the brain was dissected out. After fixation, brain tissues were immersed in 10% formalin. Then, 5 *μ*m sections were taken from the brains. The 5 sections taken from each brain were deparaffinized and stained with H&E staining. Histological analysis was performed under a light microscope, and then, images were displayed by embedding a digital camera attached to a computer monitor. Three fields were selected from each slide, and the density of dark neurons and natural neurons within the pyramidal layer of the CA3 region was estimated. The thickness of the CA3 layer was determined by a pathologist using the ImageJ software.

### 2.6. Gene Expression of NMDA Receptor Subunits

At the end of the experiment, the hippocampus was isolated and the expression of NR2A and NR2B subunits of NMDA receptor was assessed by Real-Time PCR. RNA was extracted with trizol, and cDNA synthesis was performed using a kit (Yekta Tajhiz, Iran). The PCR for each of the genes was in triplicate and repeated twice. The specific primers were designed using Primer3 Input (version 0.4.0), and H2afz gene as a normalizer was used to modify the expression level of the target genes compared to the control group. Histone H2A variant, H2afz, was used as normalizer gene, and variations in expression of each mRNA in comparison with H2afz were measured based on 2^−*ΔΔ*Ct^ relative expression formula, as described previously [33]. The sequences of primers are presented in Table 1.

### 2.7. Data Analysis

The results were analyzed by SPSS 16 software. One-way ANOVA followed by Tukey's posttest was used for multiple comparisons. Significant differences were considered at *P* < 0.05. Results were expressed as mean ± SEM.

## 3. Results

### 3.1. Rutin Decreased the Immobility Time in the FST of MS Mice

**Figure 1:**
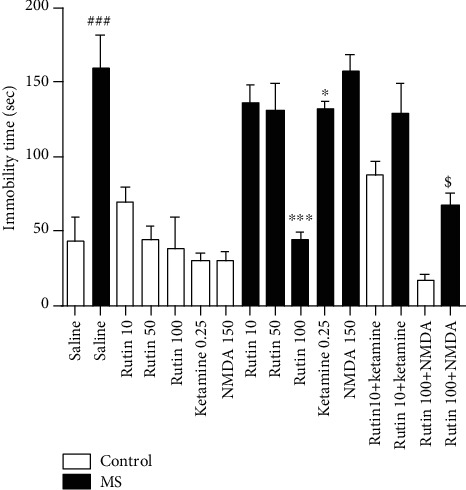
Comparison of immobilization duration in forced swimming test in experimental groups. Data are expressed as mean ± SEM (ANOVA and Tukey post hoc test). ^###^*P* < 0.001 compared with the control group (normal), and ∗*P* < 0.05 and ∗∗∗*P* < 0.001 in comparison with the MS group. ^$^*P* < 0.05 compared with the MS group which received rutin at the dose of 100 mg/kg. MS: maternal separation.

One-way ANOVA showed that there is a significant difference among the experimental groups (*F* (15, 109) = 47.13 (*P* < 0.001)). The duration of immobilization of the MS mice was significantly longer than that of the control group (*P* < 0.001). The immobility time of the MS mice which received rutin at a dose of 100 mg/kg significantly decreased in comparison with that of the MS group (*P* < 0.001). The administration of ketamine (0.25 mg/kg) significantly decreased the immobility of MS mice in comparison with the MS mice which received saline (*P* < 0.05). The duration of immobilization of the MS mice which received rutin (10 mg/kg) plus ketamine was not significantly different from that of the MS group which received rutin alone. The immobility time of the MS mice which received rutin (100 mg/kg) plus NMDA significantly increased in comparison with that of the MS group which received rutin alone (*P* > 0.05, Figure 1).

### 3.2. Rutin Modulated the Gene Expression of NMDA Receptor Subunits in the Hippocampus

**Figure 2:**
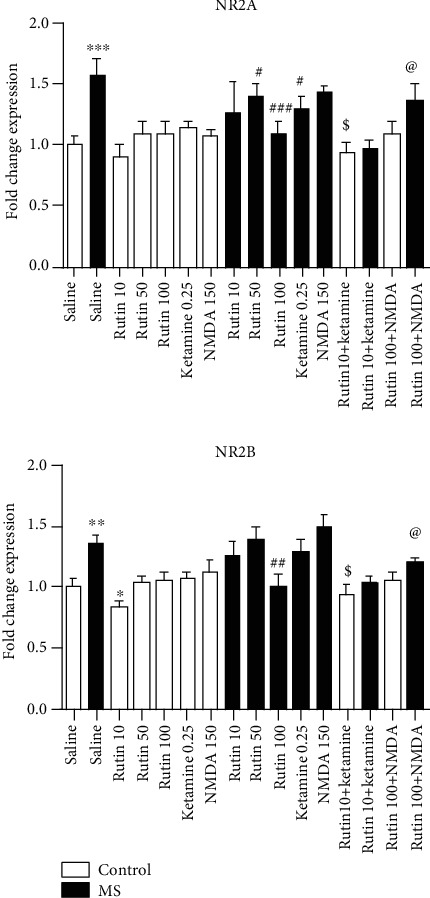
Comparison of the expression of NR2A and NR2B genes in the hippocampus. Data are expressed as mean ± SEM (ANOVA and Tukey post hoc test). ∗*P* < 0.05, ∗∗*P* < 0.01, and ∗∗∗*P* < 0.001 in comparison with the control group. ^#^*P* < 0.05, ^##^*P* < 0.01, and ^###^*P* < 0.001 in comparison with the MS group. ^$^*P* < 0.05 in comparison with the MS group which received rutin at a dose of 10 mg/kg. ^@^*P* < 0.05 in comparison with the MS group which received rutin at a dose of 100 mg/kg. MS: maternal separation.

One-way ANOVA showed that there is a significant difference among the experimental groups (*F* (15, 49) = 9.24 (*P* < 0.001)). Based on the results, the expression of NR2A subunit of NMDA receptor in the MS mice is significantly higher than that in the control group (*P* < 0.001, Figure 2). The expression of NR2A is significantly lower in the MS mice which received rutin at the doses of 50 and 100 mg/kg than in the MS group which received saline (*P* < 0.05 and *P* < 0.001). The administration of ketamine (0.25 mg/kg) significantly decreased the expression of NR2A in the MS group in comparison with the MS mice which received saline (*P* < 0.05). The expression of NR2A subunit of NMDA receptor in the MS mice which received 10 mg/kg rutin plus ketamine is significantly lower than that in the MS group which received rutin at a dose of 10 mg/kg alone (*P* < 0.05). The expression of NR2A in the MS group which received 100 mg/kg rutin and NMDA is significantly higher than that in the MS group which received rutin at a dose of 100 mg/kg rutin alone (*P* < 0.05).

One-way ANOVA showed that there is a significant difference among the experimental groups (*F* (15, 49) = 14.28 (*P* < 0.001)). The expression of NR2B subunit of NMDA receptor in MS mice is significantly higher than that in the control group (*P* < 0.01). The expression of NR2B subunit is significantly lower in the MS mice which received rutin at the dose of 100 mg/kg than in the MS group which received saline (*P* < 0.01). The expression of NR2B is significantly lower in the MS mice which received 10 mg/kg rutin plus ketamine than in the MS group which received rutin at a dose of 10 mg/kg alone (*P* < 0.05), as well as in the MS group which received 100 mg/kg rutin plus NMDA than in the MS group which received rutin at a dose of 100 mg/kg alone (*P* < 0.05).

### 3.3. Rutin Increased the Diameter of the CA3 Area of the Hippocampus

**Figure 3:**
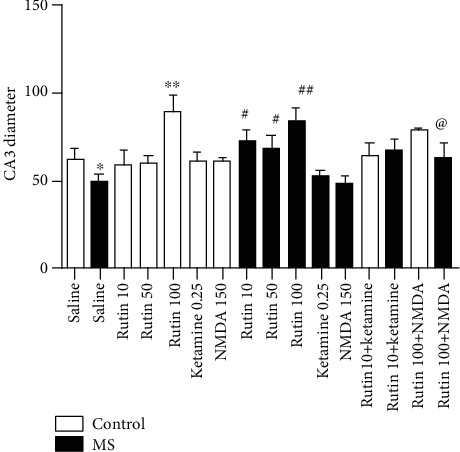
Comparison of hippocampal CA3 region diameter in experimental groups. Data are expressed as mean ± SEM (ANOVA and Tukey post hoc test). ∗*P* < 0.05 and ∗∗*P* < 0.01 in comparison with the control group. ^#^*P* < 0.05 and ^##^*P* < 0.01 in comparison with the MS group. ^@^*P* < 0.05 in comparison with the MS group which received rutin at the dose of 100 mg/kg. MS: maternal separation.

**Figure 4:**
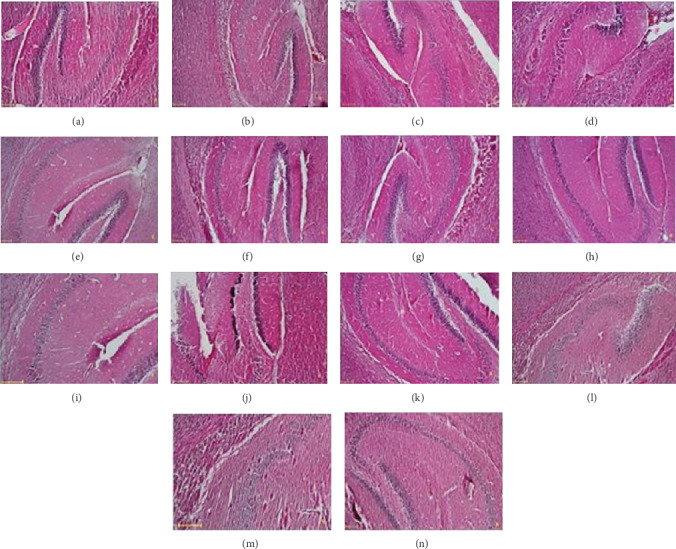
Representative features from the diameter of the CA3 region of the hippocampus pyramidal area. (a) Control, (b) MS, (c) rutin 10 (control), (d) rutin 10 (MS), (e) rutin 50 (control), (f) rutin 50 (MS), (g) rutin 100 (control), (h) rutin 100 (MS), (i) NMDA 150 (control), (j) ketamine 0.25 (control), (k) NMDA 150 (MS), (l) ketamine 0.25 (MS), (m) rutin 10+ketamine (MS), and (n) rutin 100+NMDA (MS). H&E staining (scale bar = 25 micrometer).

One-way ANOVA showed that there is a significant difference among the experimental groups (*F* (15, 48) = 10.26 (*P* < 0.001)). Based on the results (Figures 3 and 4), hippocampal CA3 diameter is significantly lower in the MS mice than in the control group (*P* < 0.05). Hippocampal CA3 region diameter is significantly higher in the MS mice which received rutin at doses of 10, 50 (*P* < 0.05), and 100 (*P* < 0.01) mg/kg than in the MS group which received saline. Hippocampal CA3 region diameter in the MS mice which received rutin (10 mg/kg) plus ketamine is not significantly different from the MS group which received rutin at a dose of 10 mg/kg alone (*P* > 0.05). Hippocampal CA3 region diameter in the MS group which received rutin (100 mg/kg) plus NMDA is significantly increased in comparison with the MS group which received 100 mg/kg rutin alone (*P* < 0.05).

### 3.4. Rutin Decreased the Percent of Dark Neurons in the Pyramidal Area of the Hippocampus

**Figure 5:**
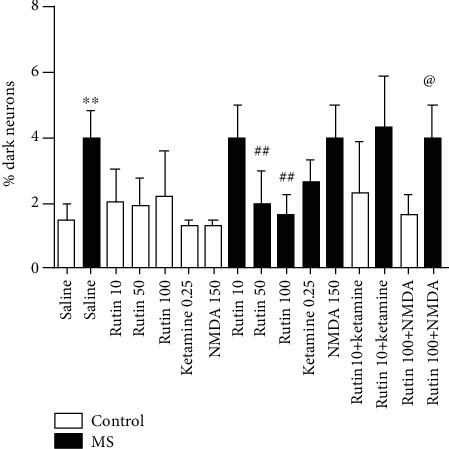
Percentage of dark hippocampal neurons in experimental groups. Data are expressed as mean ± SEM (ANOVA and Tukey post hoc test). ∗∗*P* < 0.01 in comparison with the control group. ^##^*P* < 0.01 in comparison with the MS group. MS: maternal separation.

**Figure 6:**
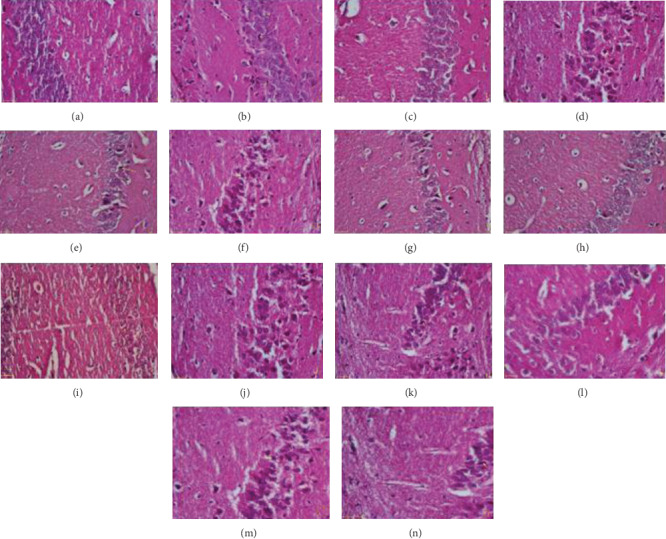
Representative features from dark neurons in the CA3 region of the hippocampus pyramidal area. (a) Control, (b) MS, (c) rutin 10 (control), (d) rutin 10 (MS), (e) rutin 50 (control), (f) Rutin 50 (MS), (g) rutin 100 (control), (h) rutin 100 (MS), (i) NMDA 150 (control), (j) ketamine 0.25 (control), (k) NMDA 150 (MS), (l) ketamine 0.25 (MS), (m) rutin 10+ketamine (MS), and (n) rutin 100+NMDA (MS). H&E staining (scale bar = 25 micrometer).

One-way ANOVA showed that there is a significant difference among the experimental groups (*F* (15, 49) = 11.21 (*P* < 0.01)). Results showed that the percentage of dark neurons in the MS mice is significantly higher than that in the control group (*P* < 0.01, Figures 5 and 6). The percentage of dark hippocampal neurons in the MS mice which received rutin at the doses of 50 and 100 mg/kg is significantly lower than that in the MS group (*P* < 0.01). Coadministration of rutin (10 mg/kg) plus ketamine in the MS group had no significant effect compared to the rutin-received counterpart (*P* > 0.05). Furthermore, coinjection of rutin (100 mg/kg) and NMDA in the MS group did not significantly change the percentage of dark hippocampal neurons in comparison with the MS group which received rutin at a dose of 100 mg/kg alone (*P* > 0.05).

## 4. Discussion

According to the results of the present study, exposure of mice at early life into a stressful condition induced by maternal separation paradigm was associated with depression-like behavior during adolescence. Maternally separated mice showed an increase in immobility time in the forced swimming test (FST). The FST is one of the most valid and common tests use for assessing depressive-like behaviors in rodents [34]. According to Seligman's theory of helplessness, if the animal is exposed to constant stress situations and has no way to escape, it gradually loses hope of escape [35]. The FST reflects one stage of desperate behavior in which depressed mice show greater immobility time [32, 36]. Previous studies have shown that reserpine-induced depression [37] and chronic stress-induced depression models are associated with an increase in the immobility time in the FST [38, 39]. Our findings are in line with aforementioned studies in which MS stress is accompanied with an increase in the immobility time in the FST in comparison with the control mice.

Previous studies have shown that flavonoids exerted neuroprotective effects and attenuated the depressive-like behaviors [40, 41]. In this regard, various pharmacological effects have been reported for rutin including neuroprotective, antioxidative stress, anti-neuroinflammatory, antidiabetic, and nephroprotective effects [42–46]. In 2017, Parashar et al. demonstrated that rutin possessed antidepressant- and anxiolytic-like effects in rats exposed to unpredictable chronic stress paradigm [11]. Yusha'u et al. showed that rutin decreased the duration of immobility in the FST in open space forced swim test model of depression in mice [47]. In the present study, we found that administration of rutin to the MS mice significantly reduced immobilization time in the FST. However, the exact mechanisms of action involved in antidepressant-like effects of rutin have not yet been established.

The hippocampus is a part of the limbic system which plays an important role in the pathophysiology of depression [9, 48, 49]. In the study by Parashar et al., exposure of mice to unpredictable chronic stress was associated with a significant increase in the hippocampal CA3 neurodegeneration [11]. Ekova et al. reported dystrophic lesions in the hippocampal CA1 and CA3 neurons in mice exposed to stressful condition [12]. In the case of clinical studies, it has been determined that brain MRI images of patients with depression show changes in hippocampal volume and density of CA1 and CA3 pyramidal neurons [10, 50]. In line with the abovementioned studies, our results showed that MS is associated with neurodegeneration in the pyramidal area of the hippocampus. We found that MS decreased the diameter of the CA3 area as well as increased the percentage of dark neurons in the hippocampus.

It has been demonstrated that rutin prevented the death and apoptosis of neurons and reduced the production of free radicals and reactive oxygen species in the brain of doxorubicin-receiving mice, suggesting neuroprotective effects for this compound [28]. Oboh et al. have reported that rutin possessed neuroprotective effects against cadmium-induced neurotoxicity [27]. Our findings showed that administration of rutin to the MS mice significantly increased the diameter of the CA3 area and decreased the percentage of dark neurons in this area. In light of the above, it seems that antidepressant-like activity of rutin probably is due to its protective effects on hippocampal CA3 neurons. However, the determination of the possible mechanisms involved in this neuroprotection has not been determined and further researches are warranted for introducing the exact mechanism of action of rutin.

Glutamate is a major primary chemical mediator in the brain [51, 52]. Glutamate's NMDA receptors are one of the important mediators of synaptic plasticity and play an important role in the neurobiological mechanisms of depression [53, 54]. Previous clinical and preclinical studies have demonstrated that administration of NMDA antagonists attenuated depressive (-like) behaviors [16, 55]. It has been determined that acute administration of ketamine possesses rapid antidepressant (-like) effects [56, 57]. In this regard, literature said that NMDA agonists provoked depressive-like behaviors in rodents [58]. Our findings showed that coadministration of NMDA receptor antagonist (ketamine) with a subeffective dose of rutin did not significantly potentiated the antidepressant-like effect of rutin. However, coadministration of an effective dose of rutin plus NMDA attenuated the antidepressant-like effect of rutin at an effective dose, indicating that NMDA to some extent mediated the antidepressant-like effect of rutin. Ample evidence demonstrated that exposure to stressful conditions increased the expression of the NR1 and NR2 subunits of the hippocampal NMDA receptor [15, 59]. Results of the present study showed that early life stress is associated with the increase in the expression of NR2A and NR2B subunits of the NMDA receptors indicating the role of NMDA receptors in the modulation of depressive-like behaviors following maternal separation paradigm. Our findings showed that rutin significantly decreased the expression of NR2B and NR2A subunits of NMDA receptor in MS mice.

## 5. Conclusion

Based on the results of the present study, rutin, partially at least, through NMDA receptors possessed an antidepressant-like effect in maternally separated mice. We showed that rutin attenuated the negative effects of MS on the hippocampal CA3 area, decreasing the number of dark neurons and increasing the diameter of this area.

## Figures and Tables

**Table 1:** Primer sequences.

Name	Sequence
Nr2A-F	CTCAGCATTGTCACCTTGGA
Nr2A-R	GCAGCACTTCTTCACATTCAT
Nr2B-F	CTACTGCTGGCTGCTGGTGA
Nr2B-R	GACTGGAGAATGGAGACGGCTA
H2afz-F	TCATCGACACCTGAAATCTAGGA
H2afz-R	AGGGGTGATACGCTTTACCTTTA

## Data Availability

We ensure that our data is available during the publishing process.
